# Triple-Frequency Electromagnetic Stimulation Combined with Fingolimod Reduces Breast Cancer Cell Proliferation and Metastasis-Associated Extracellular Vesicle Protein Levels

**DOI:** 10.3390/ph19091399

**Published:** 2026-09-04

**Authors:** Greg Haroutunian, Lawrence Daniels, Ashot Tsaghikian, Caifeng Zhao, Phaedon Zavras, Svetlana Marukian, Haiyan Zheng, Arevik Mosoian

**Affiliations:** 1 Department of Pediatrics, Keck School of Medicine, University of Southern California, Los Angeles, CA 90033, USA; ghpeds@gmail.com; 2 New Neurons LLC., Cedar Knolls, NJ 07927, USA; ldaniels@newneurons.net; 3 Data Processing and Field Engineering Corp., Glendale, CA 91214, USA; ashot.t@gmail.com; 4 Center for Advanced Biotechnology and Medicine, Rutgers University, Piscataway, NJ 08854, USA; zhaocf@cabm.rutgers.edu (C.Z.); haiyanz@cabm.rutgers.edu (H.Z.); 5 Department of Medicine, Memorial Sloan Kettering Cancer Center, New York, NY 10065, USA; zavrasp@mskcc.org; 6 FL106 Inc., c/o Flagship Pioneering, Cambridge, MA 02142, USA; smarukian@gmail.com; 7 EnergyBioCode LLC., Cedar Knolls, NJ 07927, USA

**Keywords:** electromagnetic stimulation, triple frequency, breast cancer, extracellular vesicles, TRPM7, Fingolimod (FTY720)

## Abstract

**Background:** Triple-negative breast cancer (TNBC) remains a major cause of cancer mortality due to its aggressive behavior, metabolic adaptability, and high therapeutic resistance. Extracellular vesicles (EVs) within the tumor microenvironment contribute to tumor progression and metastasis by transferring pro-tumorigenic cargo. While conventional Tumor Treating Fields use high-frequency alternating fields to disrupt mitosis, low-energy triple-frequency bioelectromagnetic approaches remain poorly characterized. **Methods:** We evaluated a device–drug strategy combining triple-frequency low-intensity electromagnetic stimulation (EMS2: 396 Hz, 285 Hz, 528 Hz) with the pleiotropic drug Fingolimod (FTY720). Treatments were tested in MDA-MB-231 and ARM-G breast cancer cells, with Paclitaxel as a positive control. Cell proliferation was assessed by MTS assay, and extracellular vesicles were isolated following individual and combination treatments. Quantitative LC-MS/MS proteomics was used to characterize treatment-induced changes in EVs cargo. **Results:** EMS2 reduced proliferation in both cell lines and produced morphological changes consistent with altered cell-cycle progression. EMS2 alone triggered adaptive metabolic responses, whereas combination with Fingolimod suppressed these compensatory signatures. EVs proteomics revealed combination-specific alterations associated with mitochondrial stress, ER stress, NF-κB suppression, and autophagy-associated pathways. The combination also reduced levels of metastasis- and stroma-associated proteins, including Mitogen-Activated Protein Kinase 12 (MAPK12) and collagen-associated ECM components (Collagen Type I Alpha 1 Chain (COL1A1), Collagen Type VI Alpha 1 Chain (COL6A1), Collagen Type VI Alpha 3 Chain (COL6A3), and Matrilin 3 (MATN3)) in EVs. Bliss independence analysis identified a subset of metastasis-associated proteins suppressed in EVs beyond the level predicted by an additive model, an exploratory finding that will require further validation with dose–response and functional assays. **Conclusions:** Combined triple-frequency EMS2 and Fingolimod treatment altered the extracellular vesicle proteome, inducing signatures consistent with mitochondrial and endoplasmic reticulum stress, metabolic disruption, and reduced levels of metastasis-associated and stromal/ECM remodeling proteins, along with reduced proliferation. These findings suggest a coordinated anti-cancer effect of this tunable device–drug strategy, warranting further functional and in vivo validation to confirm therapeutic potential.

## 1. Introduction

### 1.1. Clinical Context of Triple-Negative Breast Cancer

Triple-negative breast cancer (TNBC) represents a highly aggressive breast cancer subtype characterized by rapid progression, early metastasis, and limited therapeutic options [1,2]. Due to the absence of estrogen receptor, progesterone receptor, and HER2 expression, TNBC does not respond to targeted endocrine or HER2-directed therapies and is primarily treated with cytotoxic chemotherapy [3]. However, therapeutic resistance, tumor heterogeneity, and disease recurrence remain major clinical challenges. These limitations highlight the need for alternative strategies that can modulate tumor biology beyond direct cytotoxicity and address adaptive resistance mechanisms.

### 1.2. Extracellular Vesicle-Mediated Communication and Ion Channel Signaling in Breast Cancer

Tumor progression is strongly influenced by intercellular communication within the tumor microenvironment (TME), in which tumor-derived vesicles play a central role [4,5]. These extracellular vesicles transfer proteins, lipids, and nucleic acids that contribute to epithelial–mesenchymal transition (EMT), metastasis, immune evasion, and therapy resistance.

Among the molecular components implicated in aggressive breast cancer biology, transient receptor potential melastatin 7 (TRPM7) has emerged as a relevant ion channel associated with tumor growth, migration, and metastatic potential [6]. Elevated TRPM7 expression has been reported in breast cancer and has been linked to poor clinical outcomes. TRPM7 has also been detected in tumor-associated extracellular vesicles, suggesting a potential role in propagating pro-tumorigenic signaling within the tumor microenvironment (TME) and making it an emerging therapeutic target [7,8].

Both Fingolimod (FTY720), primarily known for its effects on sphingosine-1-phosphate signaling, and VER-155008, primarily characterized as an Hsc70/Hsp70 inhibitor, are pleiotropic compounds that have also been reported to affect TRPM7 activity [9,10]. Given their reported anti-cancer activity and the overexpression of TRPM7 in breast cancer, we selected these compounds to evaluate their effects in combination with electromagnetic stimulation.

### 1.3. Bioelectromagnetic Approaches in Cancer Therapy

Bioelectromagnetic therapies have gained increasing attention as non-invasive approaches to cancer treatment. Tumor Treating Fields (TTFields) represent a clinically validated modality that uses intermediate-frequency alternating electric fields (~200 kHz) to disrupt mitotic spindle formation and inhibit tumor cell proliferation [11,12]. TTFields have demonstrated clinical benefit in glioblastoma and are under investigation in several solid tumors, including pancreatic and ovarian cancers [11,13,14].

In parallel, low-frequency electromagnetic stimulation (EMS) has been explored in preclinical studies for its ability to influence cellular processes such as proliferation, differentiation, and membrane-associated signaling [15,16]. Building on this validated bio-electric foundation, our prior work established that fine-tuned electromagnetic stimulation (EMS) parameters can act as a tunable regulator of cell fate, directing mesenchymal stem cell differentiation [17]. Unlike high-frequency cytotoxic field-based approaches, low-energy EMS may exert subtler regulatory effects on cell behavior, although its mechanisms of action in cancer remain incompletely understood.

### 1.4. Study Rational

Calcium and magnesium signaling pathways play critical roles in cancer cell survival and proliferation, and dysregulation of ion channels, including TRP family members, has been associated with tumor aggressiveness. TRPM7 has been implicated in breast cancer progression and cellular migration [7,18,19,20].

FTY720 (Fingolimod, hereafter abbreviated FTY in tables and figures), an FDA-approved immunomodulatory drug, has been reported to exert anti-proliferative effects in cancer models through modulation of sphingosine-1-phosphate signaling and downstream survival pathways, including PI3K/AKT and mTOR, and has also been reported to affect TRPM7 activity [9,21,22,23]. Similarly, VER-155008, characterized as an Hsc70/Hsp70 inhibitor, has also been reported to affect TRPM7 activity [9,10]. Both compounds are therefore pleiotropic, with multiple pharmacological targets beyond TRPM7.

Given the involvement of ion channel signaling and metabolic adaptation in breast cancer progression, we hypothesized that combining EMS2 with pharmacological agents reported to modulate these pathways may produce complementary effects on tumor cell signaling and stress response.

### 1.5. Rationale for Combined Triple-Frequency Electromagnetic Stimulation and Pharmacological Modulation of Breast Cancer Cells

In this study, we evaluated the effects of EMS2 (396 Hz, 285 Hz, 528 Hz) alone and in combination with FTY720 in TNBC cell models (MDA-MB-231 and ARM-G) [4,24]. We assessed cellular proliferation and performed quantitative proteomic analysis of tumor-derived extracellular vesicles to investigate treatment-induced alterations in intercellular signaling.

Cancer cells exhibit distinctly different biophysical properties compared with normal cells, including altered membrane potential, ion channel activity, and intracellular signaling dynamics [25,26,27]. These differences suggest that malignant cells may be selectively sensitive to specific electromagnetic stimulation patterns, which is well established for some high-frequency alternating Tumor Treating Fields (TTFields) that disrupt mitotic spindle assembly [14,28]. Combining multiple frequencies within the high-frequency range has been shown to increase inhibitory effect over single-frequency stimulation [29,30]. Whether this holds true for the low-frequency range remains untested, since low-frequency EMS acts primarily through membrane-associated signaling and ion channel modulation rather than direct mitotic disruption. The Thomas-EMF pattern, a low-frequency complex, time-varying waveform engineered to modulate biological systems [31], exemplifies this distinct mechanism. Because low-frequency EMS operates through a fundamentally different pathway, a combination of frequencies could engage multiple complementary membrane- or channel-dependent processes simultaneously—an effect unlikely with mitotic disruption alone. This is further supported by evidence that low-frequency EMS can influence cell fate and differentiation across several stem cell lineages, including work from our own group [17], indicating a capacity for coordinated, multi-pathway reprograming. This led us to hypothesize that a structured triple-frequency combination of low-frequency EMS may offer added benefit in modulating the malignant phenotype of cancer cell lines compared with a single frequency alone.

Combining electromagnetic field therapy with chemotherapy has been reported to allow reduced chemotherapeutic dosing while maintaining efficacy [32], suggesting EMS may sensitize cancer cells to pharmacological agents. Building on this, we investigated whether low-frequency EMS combined with pharmacological modulation of ion channel and metabolic pathways could enhance anti-cancer activity beyond either approach alone.

We therefore investigated a triple-frequency combination in the low-frequency range (396–285–528 Hz), alone and in combination with a pharmacological agent reported to target multiple signaling pathways, including TRPM7, and profiled the resulting extracellular vesicle proteome in breast cancer cells as a sensitive, indirect readout of treatment-induced signaling and stress adaptation.

## 2. Results

### 2.1. Evaluation of Triple-Frequency Electromagnetic Stimulation (EMS) on Breast Cancer Cell Proliferation

To identify bioactive frequency ranges, we performed exploratory frequency screening. Initial testing at reference frequencies (50 Hz, 100 Hz, and 1000 Hz) produced no detectable activity. We then systematically explored the low-frequency range (250–550 Hz), testing frequencies at 21, 285, 396, 417 and 528 Hz. This range was selected based on published pulsed electromagnetic field (PEMF) literature describing biological activity within this window [15,33,34,35].

To reduce cellular adaptation and desensitization, a known limitation of constant-frequency stimulation in neurostimulation and PEMF therapy, we developed sequential stimulation protocols that alternate among three frequencies within the bioactive window (10 min each; 30 min total).

EMS1: 396 Hz–10 min, 417 Hz–10 min, 528 Hz–10 min;

EMS2: 396 Hz–10 min, 285 Hz–10 min, 528 Hz–10 min;

EMS3: 21 Hz–30 min.

**Figure 1:**
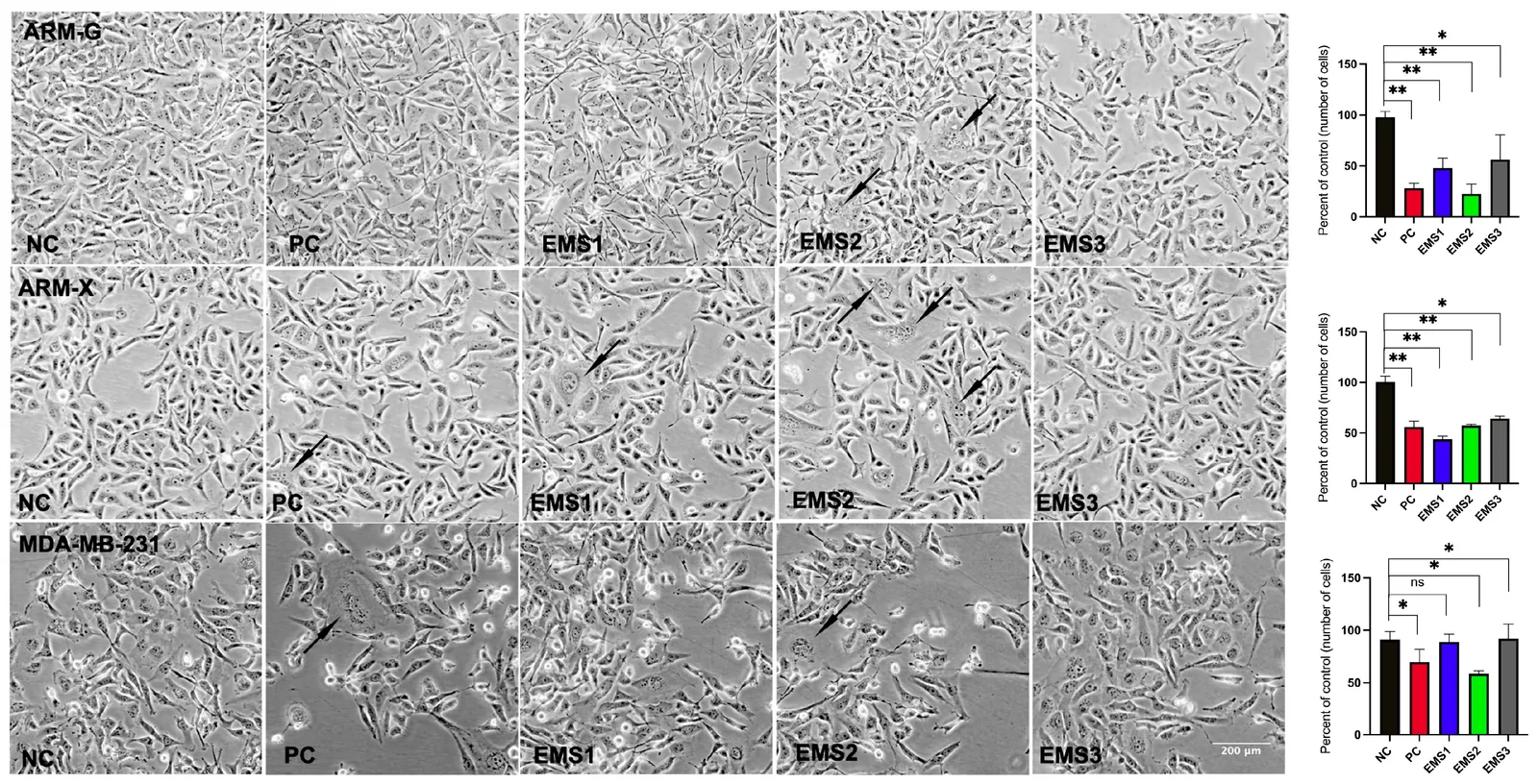
Phase-contrast microscopy images of ARM-G, ARM-X and MDA-MB-231 breast cancer cells treated with positive control (PC) EMS1, EMS2, and EMS3. ARM-G breast cancer cells treated with 25 nM Paclitaxel (positive control) or exposed to EMS1, EMS2, or EMS3. Effects of the same treatments on ARM-X breast cancer cells. Effects on the MDA-MB-231 triple-negative breast cancer cell line. Representative phase-contrast micrographs show cell morphology under the indicated conditions. Arrows indicate enlarged cells with mitotic morphology that failed to complete cell division. Graphs show percent inhibition of proliferation relative to untreated controls. Data shown are from one representative experiment (of three independent experiments), presented as mean ± SD of technical replicates. Statistical significance was determined by Student’s *t*-test. ** *p* < 0.01; * *p* < 0.05 indicate statistically significant differences compared with untreated controls; ns, not significant. Scale bar = 200 μm, calibrated based on the pixel-to-micrometer ratio of the Nikon imaging system using Fiji software (version: 2.16.0/1.54p). Representative phase-contrast micrographs are shown from one experiment, representative of 3 independent experiments with similar results.

To determine whether the physical presence of the electromagnetic coil influenced cell proliferation independently of electromagnetic stimulation, cells were exposed to a sham condition in which breast cancer cells in the tube were placed inside the coil while the device remained disconnected from the power supply for 30 min [17]. Cancer cell proliferation did not differ significantly between sham-exposed cultures and basal medium controls, indicating that the inactive apparatus had no detectable biological effect (Figure S1). Accordingly, the standard medium control was used as the negative control in all subsequent optimization experiments and paclitaxel (25 nM) served as the positive control (PC). All three EMS treatments inhibited proliferation of the ARM-G and ARM-X cell lines, although the magnitude varied by frequency and cell line (Figure 1, rows 1 and 2). We next evaluated whether EMS treatments could inhibit a second triple-negative model, MDA-MB-231, cultured in 10% serum.

Unlike ARM-G and ARM-X, the MDA-MB-231 cells responded only to EMS2, which reduced proliferation to the range of 22.5–58.7% of control. Neither EMS1 nor EMS3 produced statistically significant effects in this line (Figure 1). Three EMS protocols were screened for anti-proliferative activity, as shown in Figure 1 and the corresponding numbers in Table S1. Sequential protocols (EMS1 and EMS2) have achieved more pronounced growth inhibition in comparison with the single-frequency stimulation (EMS3) in both cancer cell lines. EMS2 was selected for subsequent combination studies based on its superior efficacy in ARM-G and MDA-MB-231 cells.

Morphological indicators are consistent with altered cell-cycle progression. Arrows indicate cells exhibiting morphological changes. Arrows indicate cells exhibiting characteristic features consistent with altered cell-cycle progression, including pronounced cell enlargement, flattened morphology, and multinucleation. This is consistent with altered cell-cycle progression and a senescence-like state, as previously reported [36,37] (Figure 1, arrows). In contrast, only sporadic enlarged cells were detected in untreated controls, indicating that this phenotype is specifically associated with EMS exposure. The increase in these abnormal cells is consistent with a mitotic blockade and/or dysregulation of cell-cycle progression.

Across the three lines, proliferation following EMS2 decreased to the range of 22.5–58.7% of control (as shown in Figure 1 and the corresponding numbers in Table S1). Treatment values are expressed as % of control; lower values indicate greater inhibition, approaching the magnitude of inhibition observed with paclitaxel (Figure 1). Based on this screening data, EMS2 was selected for subsequent proteomic characterization and combination studies with FTY720.

### 2.2. EMS2 Does Not Inhibit the Growth of Primary Human Mammary Epithelial Cells (HMECs)

**Figure 2:**
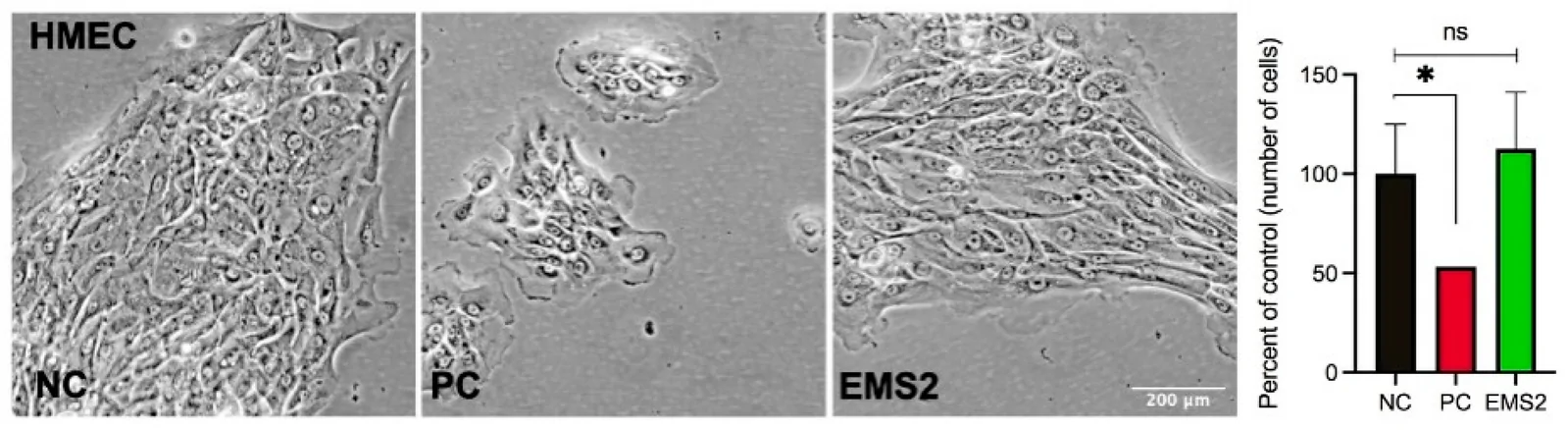
EMS2 does not inhibit proliferation of normal mammary epithelial cells: phase-contrast microscopy and proliferation analysis. HMECs were treated with 25 nM Paclitaxel (positive control) or exposed to EMS2. Phase-contrast micrographs were captured using a Nikon microscope (Nikon, Tokyo, Japan) at 200× magnification with Swift Imaging 3.0 software (Swift Optical Instruments/Motic Swift Line, Schertz, TX, USA), showing cell morphology under the indicated conditions. Graphs show percent inhibition of proliferation relative to untreated controls. Data are presented as mean ± SD of a single representative experiment (of three independent experiments), performed in triplicate. * *p* < 0.05 indicates a statistically significant difference compared with untreated controls; ns, not significant. Scale bar = 200 μm, calibrated using Fiji software (version: 2.16.0/1.54p) based on the pixel-to-micrometer ratio of the imaging system. Representative phase-contrast micrographs are shown from one experiment, representative of *n* = 3 independent experiments with similar results.

To evaluate the selectivity of EMS2 for malignant cells, we tested the effect of EMS2 on primary human mammary epithelial cells (HMECs). EMS2 did not inhibit HMEC proliferation (Figure 2), suggesting differential sensitivity between the tested malignant and non-malignant mammary cell models. This selectivity is consistent with known differences in ion-channel signaling between malignant and normal cells; however, causality has not yet been established. This finding is consistent with published data from Smothers et al., 2023 [38].

### 2.3. Fingolimod Reduces Proliferation Across All Three Breast Cancer Models

In breast cancer, TRPM7 is markedly overexpressed in high-grade (Grade III) tumors and invasive ductal carcinomas, where its expression independently predicts poor outcome and a higher risk of distant metastasis [19,39]. In prior work, we confirmed high TRPM7 expression in ARM-X and ARM-G cells. Fingolimod (FTY720) and VER-155008 are pleiotropic compounds with multiple reported pharmacological targets and documented anti-cancer activity; among their various reported activities, effects on TRPM7 have also been described [9,23,40]. Given their anti-cancer activity and the elevated TRPM7 expression observed in our cell models, we tested these compounds—VER-155008 (4 µM) [9,40] and FTY720 (5 µM) [23,40,41]—in MDA-MB-231, ARM-G, and ARM-X cells [24]. Both compounds independently reduced proliferation of the triple-negative cells (Figure 2), consistent with previously published work [42,43,44].

Among the EMS protocols, only EMS2 inhibited proliferation of MDA-MB-231, which we attribute to an interaction between its specific electromagnetic parameters (frequency and intensity) and serum-driven signaling. Under serum-rich conditions, MDA-MB-231 cells depend heavily on growth-factor signaling, and EMS2 appears to fall within a biologically active window that disrupts these pathways, consistent with other reports that serum conditions modulate EMS bioactivity [34].

The plasma concentration of FTY720 in humans is reported to be approximately 0.05–0.3 µM [45]. We utilized FTY720 concentrations of 5 µM, 0.5 µM, and 0.05 µM in subsequent experiments to assess its anticancer activity and potential additive effects when combined with EMS2 (Figure 2).

**Figure 3:**
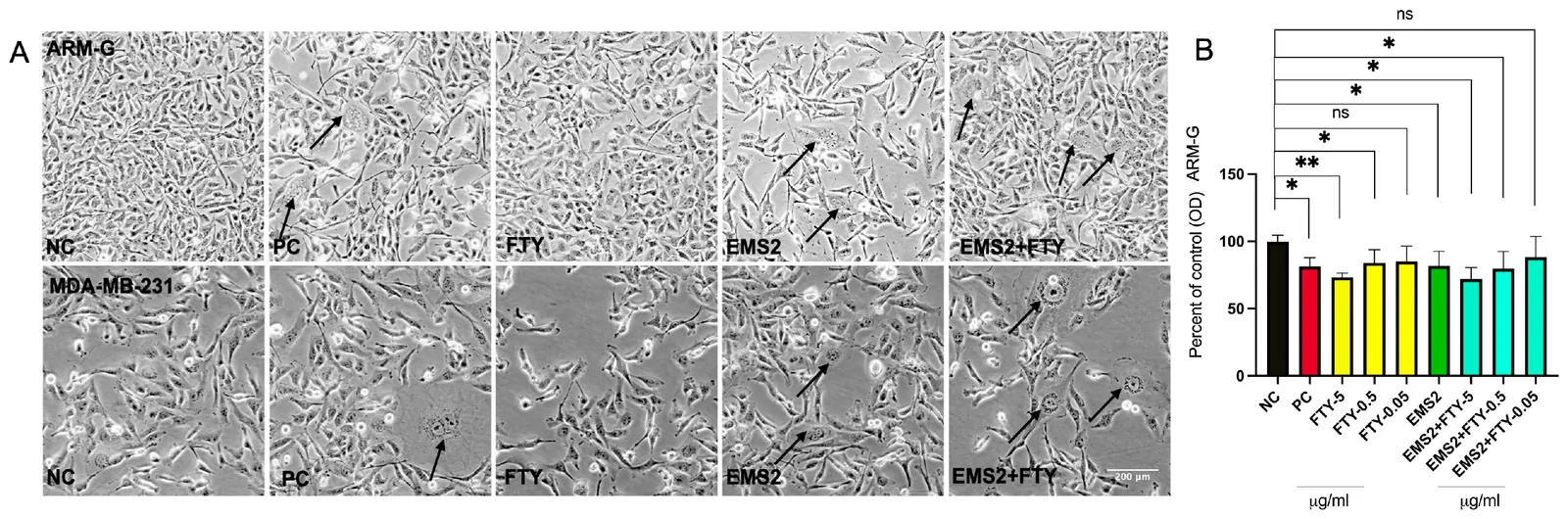
Phase-contrast microscopy images of ARM-G and MDA-MB-231 breast cancer cells treated simultaneously with EMS2 and 5 μM FTY720 for 48 h. (**A**) Upper panel: Representative phase-contrast micrographs of ARM-G breast cancer cells treated with 25 nM Paclitaxel (positive control), EMS2 alone, or 5 μM FTY720 with or without EMS2. Lower panel: Representative phase-contrast micrographs showing the effects of the same treatments on MDA-MB-231 cell morphology. Images shown are representative of *n* = 3 independent experiments with similar results. Arrows indicate cells exhibiting pronounced enlargement and flattened morphology—features consistent with altered cell-cycle progression, as previously reported (2, 3). Images were captured at 200× magnification. Scale bar = 200 μm (calibrated via Fiji software (version: 2.16.0/1.54p)). (**B**) Percent inhibition of proliferation relative to untreated controls in ARM-G cells, evaluating the combined effects of EMS2 with increasing concentrations of FTY720. Data are presented as mean ± SD of a single representative experiment (of three independent experiments), performed in triplicate. * *p* < 0.05, ** *p* < 0.01 indicate statistically significant differences compared with untreated controls; ns, not significant.

Combining FTY720 with EMS2 did not enhance proliferation inhibition beyond that achieved by either treatment alone, indicating no additive benefit at the proliferation level (Figure 3). Dose-dependent effects of FTY720 alone and in combination with EMS2 on breast cancer cell viability are shown in Figure 3 (exact values provided in Supplementary Table S2).

### 2.4. Extracellular Vesicle Isolation from Differentially Treated ARM-G Breast Cancer Cell Supernatant

ARM-G cells were selected for extracellular vesicle isolation because they grow in serum-free, supplemented medium [24], whereas MDA-MB-231 requires medium containing 10% fetal bovine serum (FBS). Because serum is itself a major source of extracellular vesicles, its presence would confound the *extracellular vesicle* proteomic profiles; the serum-free ARM-G system ensured that treatment-induced cargo changes could be attributed to extracellular vesicles released by the cancer cells in response to treatment, rather than to extracellular vesicles (EVs) derived from FBS.

**Figure 4:**
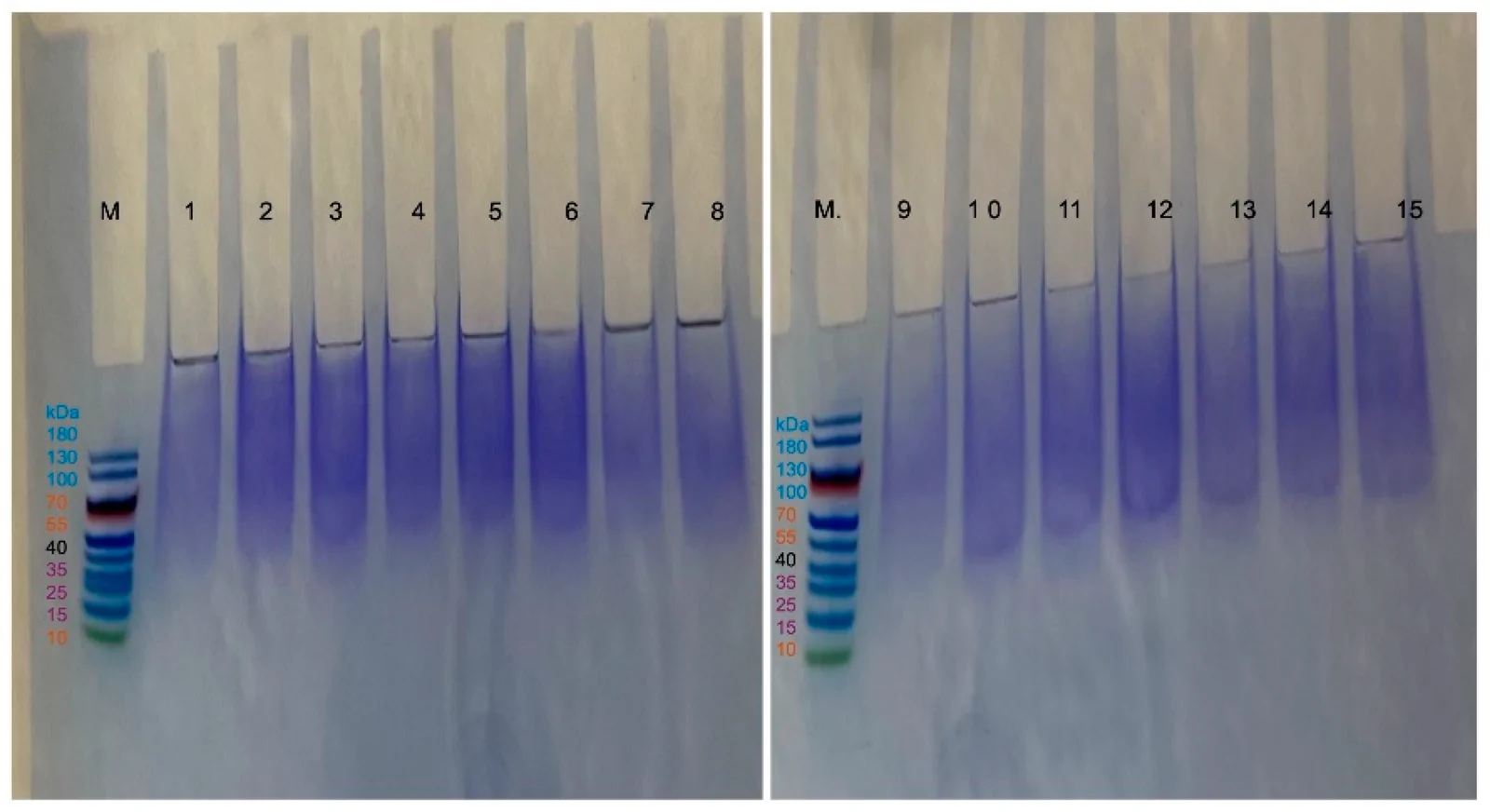
Protein profiles of EVs isolated from ARM-G breast cancer cells under different treatment conditions. ARM-G cells were cultured under the following conditions: NC, negative control (lanes 1–3); PC, positive control (25 nM Paclitaxel; lanes 4–6); 5 μM FTY720 (lanes 7–9); EMS2 + FTY720 (lanes 10–12); and EMS2 alone (lanes 13–15). EVs proteins from culture supernatants were separated by SDS–PAGE under reducing conditions and stained with Coomassie Brilliant Blue G-250, showing distinct protein banding patterns across treatment groups. M, molecular weight marker.

SDS-PAGE analysis of EVs protein content from differentially treated ARM-G cells is shown in Figure 4. LC-MS/MS-based proteomic analysis was performed at Rutgers University. Analysis confirmed high expression of the canonical extracellular vesicle markers CD81, CD63, and CD9 (Table S3), confirming that EVs identity was maintained following EMS2, FTY720, and combination treatment.

**Figure 5:**
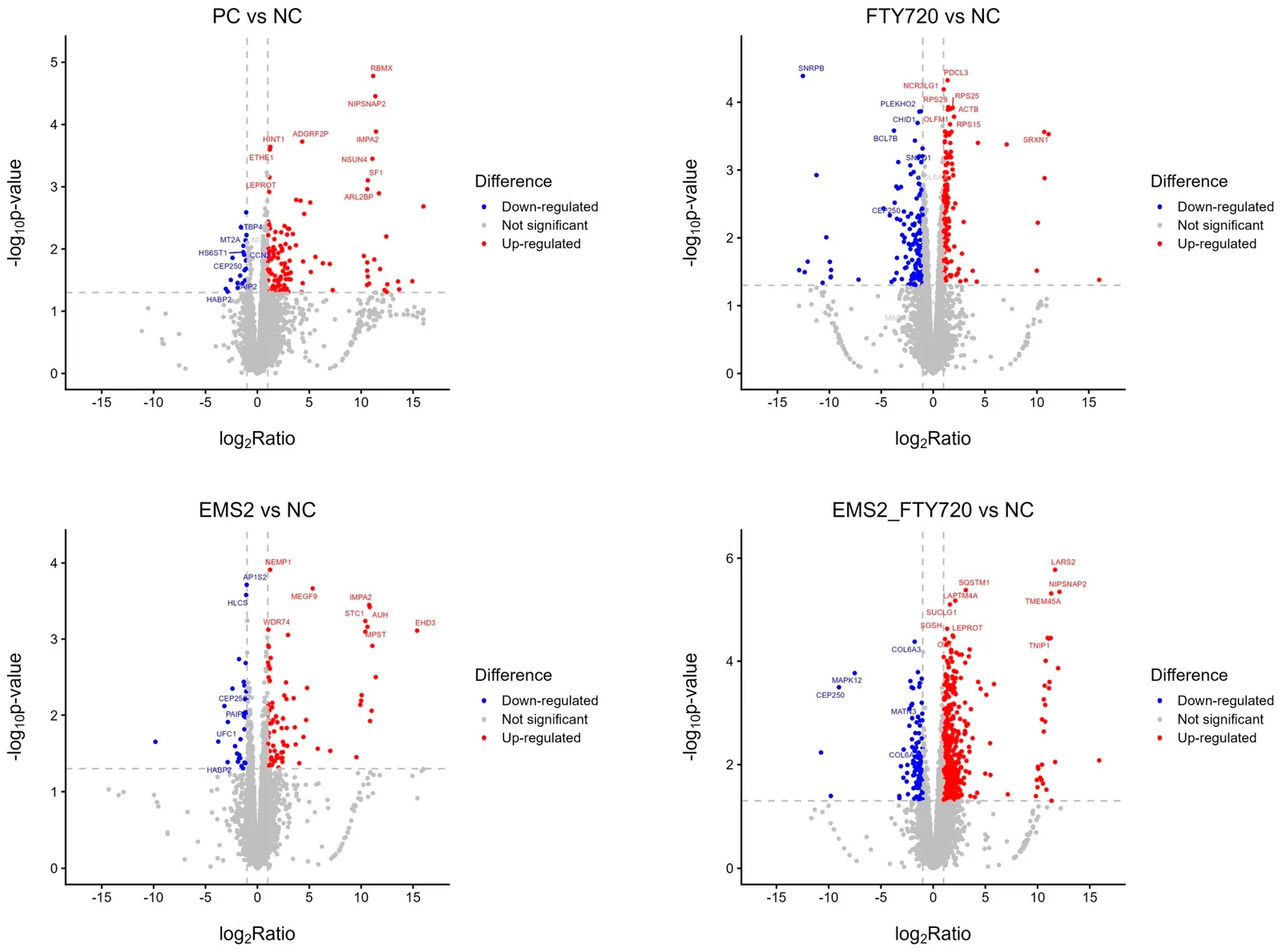
Differential extracellular vesicle protein expression displayed as volcano plots across treatment conditions. Comparisons include negative control (NC), positive control (PC), single treatments (EMS2 or Fingolimod), and the combined EMS2 with FTY720 treatment. The horizontal dashed line represents the significance threshold (*p* = 0.05), and the vertical dashed lines represent the fold-change cutoff (log2 Ratio = ±1). Red points indicate significantly up-regulated genes (log2 Ratio > 1, *p* < 0.05), and blue points indicate significantly down-regulated genes (log2 Ratio < −1, *p* < 0.05); grey points are not statistically significant.

The larger number of significant protein changes observed for the dual treatment (FTY720 + EMS2 vs. NC; Figure 5 volcano plots) is consistent with a combined effect, which we further evaluated quantitatively below.

#### 2.4.1. Paclitaxel Induces an Extracellular Vesicle Proteomic Signature Consistent with Mitotic Stress, Translational Suppression, and Metabolic Reprograming

As a positive control, cells were treated with paclitaxel, a clinically established microtubule-stabilizing chemotherapeutic with well-characterized antiproliferative activity. Proteomic profiling of EVs derived from paclitaxel-treated cells revealed a distinct molecular signature enriched in proteins associated with translational regulation, RNA modification, membrane remodeling, mitochondrial stress, and markers consistent with altered cell-cycle progression.

Membrane and vesicular remodeling. Ring Finger Protein 24 (RNF24) was detected exclusively following paclitaxel treatment (*p* = 0.01), whereas Leptin Receptor Overlapping Transcript (LEPROT) was significantly increased (log2 ratio = 1.2, *p* = 0.0007), consistent with altered membrane trafficking and EV biogenesis.

Translational and epitranscriptomic regulation. The RNA-processing proteins Nucleolar Protein- NOP2/Sun RNA Methyltransferase 4 (NSUN4) and RNA Binding Motif Protein, X-Linked (RBMX) were detected exclusively in paclitaxel-treated EVs and were absent from untreated controls (NSUN4, *p* = 0.0003; RBMX, *p* < 0.00001), consistent with treatment-induced changes in the packaging or expression of epitranscriptomic regulatory machinery.

Metabolic and mitochondrial stress. Inositol Monophosphatase 2 (IMPA2) was detected only in paclitaxel-treated EVs (*p* = 0.00001). NIPSNAP Homolog 2 (NIPSNAP2), a mitochondrial protein implicated in mitophagy, was likewise detected exclusively following treatment (*p* < 0.00001). The mitochondrial protein Ethylmalonic Encephalopathy 1 (ETHE1) was significantly increased (log2 ratio = 1.2, *p* = 0.0002), and Histidine Triad Nucleotide-binding protein 1 (HINT1) was similarly increased (log2 ratio = 1.2, *p* = 0.0002). Collectively, these EV cargo changes are consistent with activation of mitochondrial and metabolic stress-response pathways in the donor cell, though direct functional assays would be required for confirmation.

Altered cell-cycle progression and translational suppression. Paclitaxel significantly reduced the Centrosomal Protein 250 (CEP250) (log2 ratio = −2.4, *p* = 0.01) and the adhesion-associated protein Hyaluronan Binding Protein 2 (HABP2) (log2 ratio = −2.9, *p* = 0.04) in extracellular vesicle cargo, consistent with disruption of centrosome integrity and impaired cell adhesion in the parent cell; however, as these proteins were measured in EVs rather than in treated cells directly, changes in EV abundance may also reflect altered cargo sorting or vesicle biogenesis rather than a one-to-one representation of intracellular protein levels. Tissue Factor Pathway Inhibitor (TFPI) was also significantly decreased (log2 ratio = −1.9, *p* = 0.03); given its established role in coagulation regulation rather than mitosis or translation, this change may reflect broader remodeling of extracellular vesicle cargo composition and warrants further investigation. Poly(A) Binding Protein Interacting Protein 2 (PAIP2), a translational repressor that competes with eIF4G for PABP binding, was significantly reduced in EV cargo (log2 ratio = −2.6, *p* = 0.03); rather than suppressing protein synthesis directly, this decrease would be expected to relieve translational repression and may instead reflect a compensatory response within the broader proteotoxic stress program of the donor cell. Increased EV abundance of the stress-response chaperones Heat Shock Protein Family A (Hsp70) Member 1A/Member 1B (HSPA1A/B) and BCL2 Associated Athanogene 3 (BAG3) is consistent with activation of a proteotoxic stress response in the donor cell, although functional assays in treated cells—rather than EV cargo analysis alone—would be needed to confirm pathway activation.

Collectively, these EV cargo changes reveal a proteomic signature consistent with paclitaxel-induced mitotic stress and mitochondrial stress in the donor cells, alongside altered membrane and vesicular remodeling, supporting paclitaxel’s use as a positive control. Because these inferences are based on extracellular vesicle content rather than direct analysis of treated cells, and because functional assays were outside the scope of this study, they should be interpreted as indirect, hypothesis-generating evidence rather than direct confirmation of intracellular mitotic or metabolic status.

#### 2.4.2. FTY720 Induces an Extracellular Vesicle Proteomic Signature Consistent with Ribosomal Stress, Oxidative Response, and Extracellular Matrix Remodeling

To characterize the molecular effects of FTY720, EVs isolated from treated ARM-G breast cancer cells were analyzed by LC-MS/MS. FTY720 induced a distinct proteomic signature enriched in proteins associated with ribosomal stress, oxidative stress responses, immune signaling, and extracellular matrix (ECM) remodeling.

Ribosomal and translational stress. FTY720 significantly increased the abundance of Ribosomal Proteins S29, S25 and S15; RPS29 (log2 ratio = 1.5, *p* < 0.00001), RPS25 (log2 ratio = 1.9, *p* < 0.00001), and RPS15 (log2 ratio = 2.0, *p* < 0.00001) in EVs cargo, consistent with disruption of translational homeostasis and activation of nucleolar stress pathways in the donor cell.

Oxidative stress and cellular adaptation. FTY720 was associated with a proteomic signature consistent with an oxidative stress response, highlighted by increased expression of Heme Oxygenase 1 (HMOX1)(log2 ratio = 2.2, *p* < 0.00001) and Sulfiredoxin 1 (SRXN1)(log2 ratio = 1.8, *p* = 0.0003), both established Nrf2-responsive antioxidant proteins. Additional proteins showing increased abundance included Phosducin Like 3 (PDCL3)(log2 ratio = 1.4, *p* < 0.00001) and Olfactomedin 1 (OLFM1)(log2 ratio = 1.4, *p* = 0.0001); while these are not classical oxidative stress markers, their co-elevation alongside Heme Oxygenase 1 (HMOX1) and Sulfiredoxin 1 (SRXN1) may reflect broader cellular stress adaptation. Actin Beta (ACTB) was also increased (log2 ratio = 1.7, *p* = 0.0001); as a commonly used loading-control protein, this change should be interpreted cautiously and confirmed independently of normalization procedures before being attributed to a genuine stress response.

Immune-related signaling. The natural killer cell ligand Natural Killer Cell Cytotoxicity Receptor 3 Ligand 1 (NCR3LG1)(B7-H6) was significantly upregulated (log2 ratio = 1.0, *p* < 0.00001) in EVs; given B7-H6′s established role as an NK cell-activating ligand, this change is consistent with a proteomic signature associated with tumor immunogenicity, though direct assessment of NK cell recognition or cytotoxicity would be required to confirm functional susceptibility [46,47,48]. Endonuclease Domain Containing 1 (ENDOD1), a negative regulator of the cGAS-STING innate immune signaling pathway, was detected only in FTY720-treated EVs (*p* = 0.001), consistent with treatment-associated changes in innate immune signaling-related cargo.

Extracellular matrix remodeling and suppression of invasion-associated signaling pathways. FTY720 significantly reduced multiple proteins associated with extracellular matrix organization and tumor invasion in EV cargo. Tenascin-C (TNC) and Sushi, Nidogen and EGF-Like Domains 1 (SNED1) were downregulated (both log2 ratio = −2.2, *p* = 0.0008), consistent with a signature associated with disruption of the metastatic niche. Additional decreases were observed for Pleckstrin Homology Domain Containing O2 (PLEKHO2)(log2 ratio = −1.2, *p* = 0.0001), Chitinase Domain Containing 1 (CHID1)(log2 ratio = −1.3, *p* = 0.0001), and BCL Tumor Suppressor 7B (also known as B-Cell CLL/Lymphoma 7B, BCL7B)(log2 ratio = −3.8, *p* = 0.0002), consistent with suppression of signaling pathways associated with tumor progression and invasion, pending functional confirmation. Small Nuclear Ribonucleoprotein Polypeptides B and B1 (SNRPB), a core spliceosomal component, was detected in control EVs but was absent following FTY720 treatment (*p* < 0.00001); given its role in RNA splicing rather than ECM biology, this change is reported as a presence–absence event of uncertain functional significance in this context rather than evidence of ECM remodeling.

Summary proteomic signature. Collectively, FTY720 treatment produced a coordinated extracellular vesicle proteomic signature characterized by markers associated with ribosomal and translational stress, oxidative stress responses, altered innate and adaptive immune-related signaling, and suppression of extracellular matrix organization and invasion-associated pathways [43]. These findings are consistent with disruption of translational homeostasis, impaired matrix-remodeling capacity, and activation of cellular stress and immune-recognition pathways in the donor cells. Because these inferences are based on EV cargo rather than direct functional assays, they should be interpreted as hypothesis-generating evidence of a potential inhibitory effect on breast cancer progression, rather than direct confirmation of tumor vulnerability or reduced malignant potential.

#### 2.4.3. EMS2 Treatment Induces an Extracellular Vesicle Proteomic Signature Consistent with Oxidative Stress Response, Metabolic Adaptation, and Structural Remodeling

To characterize the effects of electromagnetic stimulation alone, LC-MS/MS proteomic profiling was performed on EVs isolated from EMS2-treated ARM-G breast cancer cells. EMS2 was associated with a distinct proteomic profile characterized by coordinated changes in proteins associated with oxidative stress, mitochondrial metabolism, vesicular trafficking, and structural organization.

Oxidative stress and metabolic adaptation. EMS2 treatment significantly enriched several proteins involved in oxidative stress defense and mitochondrial metabolism. AU RNA Binding Methylglutaconyl-CoA Hydratase (AUH), Inositol Monophosphatase 2 (IMPA2), and Mercaptopyruvate Sulfurtransferase (MPST) were detected exclusively in EMS2-treated EVs and were absent from untreated controls (AUH, *p* = 0.0003; IMPA2, *p* = 0.0003; MPST, *p* = 0.0006) and are therefore reported as presence–absence regulated features rather than quantitative fold changes. Leucyl-tRNA Synthetase 2, Mitochondrial- LARS2 also showed a quantitative increase (log2 ratio = 1.0, *p* = 0.001), consistent with altered mitochondrial translational activity.

Vesicular trafficking and extracellular vesicle remodeling. EMS2 also induced exclusive detection of proteins associated with endosomal transport and extracellular vesicle dynamics: EH Domain Containing 3 (EHD3) (*p* = 0.0007), Galectin 7 (LGALS7) (*p* = 0.003), and Stanniocalcin 1 (STC1) (*p* = 0.0005). None of these three proteins were detected in control EVs, consistent with treatment-associated changes in EVs cargo export.

Structural organization and cell-cycle regulation. Parallel EVs proteomic analysis demonstrated significant downregulation of proteins involved in centrosome integrity, chromosome organization, autophagy, and proteostasis. CEP250 (log2 ratio = −3.18, *p* = 0.007) and Chromosome Alignment Maintaining Phosphoprotein 1 (CHAMP1) (log2 ratio = −2.1, *p* = 0.02) were reduced, consistent with disrupted centrosome and chromosome organization, respectively. RB1 Inducible Coiled-Coil 1 (commonly known as FIP200)—RB1CC1/FIP200 (log2 ratio = −2.4, *p* = 0.004), a core autophagy regulator, and Ubiquitin-Fold Modifier Conjugating Enzyme 1 (UFC1) (log2 ratio = −3.7, *p* = 0.02), a component of the UFMylation pathway involved in ribosome-associated quality control and ER stress response, were also significantly reduced, consistent with broader disruption of proteostatic and quality-control mechanisms. PAIP2 (log2 ratio = −2.4, *p* = 0.004), a translational repressor, was likewise decreased; rather than directly suppressing translation, this reduction would be expected to relieve repression of cap-dependent translation and may instead reflect a compensatory response within the broader stress program.

Extracellular matrix remodeling and signaling. EMS2 treatment also altered proteins associated with extracellular organization and signaling. Multiple EGF Like Domains 9- MEGF9 was detected exclusively in EMS2-treated EVs and was absent from untreated controls (*p* = 0.0002) and is therefore reported as a presence–absence regulated feature rather than a quantitative fold change. Serpin Family A Member 3 (SERPINA3) was detected in control EVs but was absent following EMS2 treatment (*p* = 0.02) and is likewise reported as a presence–absence event rather than a quantitative fold change, given the extreme magnitude of the apparent fold difference. Hyaluronan Binding Protein 2-HABP2 was significantly downregulated (log2 ratio = −2.8, *p* = 0.04). RAS P21 Protein Activator 4 (RASA4) was increased (log2 ratio = 2.5, *p* = 0.02); RASA4 has been reported to suppress proliferation via inhibition of HIF-α signaling in other cancer contexts (cervical cancer) [49], suggesting a possible role in modulating proliferative signaling in this system, though this inference is drawn from a different cancer type and has not been functionally tested here.

##### EMS2 Treatment Elicits a Proteomic Signature Consistent with a Senescence-like State

EMS2 treatment induced a proteomic signature consistent with a senescence-like state [50]. This includes the downregulation of key cell-cycle and quality-control regulators (CEP250, CHAMP1, RB1CC1, and UFC1) alongside the upregulation of RASA4 and the exclusive EV detection of STC1 and LGALS7, proteins previously associated with the senescence-associated secretory phenotype (SASP) [51,52]. While these proteomic changes are consistent with known senescence-associated pathways, confirming a true senescent phenotype would require dedicated functional assays—including SA-β-gal staining, cell-cycle profiling, and p53/p21 or p16/Rb pathway assessment—which were outside the scope of this study.

##### Summary Proteomic Signature

Collectively, EMS2 treatment was associated with coordinated changes in extracellular vesicle proteins involved in oxidative stress responses, mitochondrial metabolism, vesicular trafficking, structural organization, extracellular matrix regulation, and markers associated with senescence-like state. The enrichment of stress-response and vesicular export proteins, together with the reduction in proteins controlling centrosome integrity, chromosome organization, autophagy, and proteostasis, is consistent with disrupted structural and quality-control homeostasis. Parallel changes in extracellular matrix-associated proteins and increased RASA4—previously linked to suppressed proliferation via HIF-α signaling inhibition in other cancer contexts [49]—are consistent with, but do not establish, a shift toward reduced proliferative or invasive capacity following EMS2 treatment; functional validation in this system would be required to confirm these effects.

#### 2.4.4. Combined EMS2 and FTY720 Treatment Induces an Extracellular Vesicle Proteomic Signature Consistent with Coordinated Stromal Remodeling, Autophagy-Pathway Alteration, and Metabolic Stress

To investigate whether electromagnetic stimulation enhances the anticancer activity of TRPM7 inhibition, LC-MS/MS proteomic profiling was performed on EVs isolated from ARM-G cells treated with the combined EMS2 + FTY720 regimen. Compared with either monotherapy, the combination induced the largest number of differentially expressed proteins, indicating extensive proteomic remodeling.

**Figure 6:**
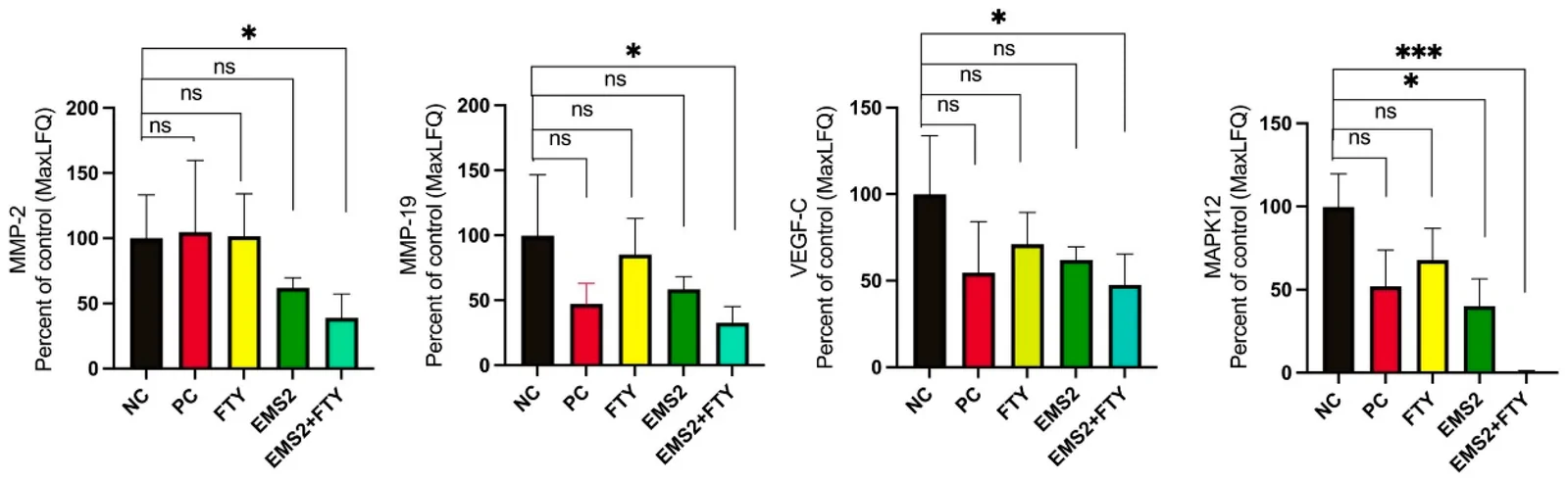
Combined EMS2 and FTY720 treatment suppresses metastasis-associated factors beyond the Bliss-predicted expectation in breast cancer cell line ARM-G. MMP-2, MMP-19, VEGF-C, and MAPK12 protein levels were significantly reduced following combination treatment. Data represent MaxLFQ normalized intensities and are presented as mean ± SD. * *p* < 0.05, *** *p* < 0.001; ns, not significant (compared with untreated controls).

Treatment-induced suppression of metastasis-associated protein levels in EVs. To validate the proteomic findings, we quantified several established mediators of tumor invasion and metastasis. Relative to untreated controls, the combined treatment reduced Matrix Metalloproteinase-2 (MMP-2) by 66.0%, Matrix Metalloproteinase-19 (MMP-19) by 61.0%, Vascular Endothelial Growth Factor-C (VEGF-C) by 48.0%, and MAPK12 by 99.5% (Figure 6). Neither EMS2 nor FTY720 alone produced statistically significant inhibition of these proteins. Bliss independence analysis of MMP-2, MMP-19, VEGF-C, and MAPK12 demonstrated greater inhibition than predicted from the individual treatments, representing a greater-than-additive suppression effect (Table 1).

Combined treatment with EMS2 and FTY720 produced greater-than-additive downregulation of all four proteins examined (MMP-2, MMP-19, VEGF-C, and MAPK12), with observed combination values lower than the Bliss-predicted expected values (E_Bliss) in each case (Table 1*).* The treatment reduces the abundance of EV-associated proteins previously implicated in metastasis and invasion, suggesting a potential down-regulation that warrants functional validation.

Extracellular matrix and stromal remodeling. The most prominent proteomic signature of the combined treatment was extensive remodeling of extracellular matrix components. Major fibrillar and basement membrane collagens were significantly reduced, including Collagen type I Alpha 1 chain (COL1A1)(log2 ratio = −2.2, *p* = 0.0002), Collagen type IV Alpha 2 chain (COL4A2)(log2 ratio = −1.2, *p* = 0.017), Collagen type VI Alpha 1 hain (COL6A1)(log2 ratio = −2.0, *p* = 0.004), and Collagen type VI Alpha 3 chain (COL6A3) (log2 ratio = −2.0, *p* < 0.00001). The extracellular matrix cross-linking protein Matrilin 3 (MATN3) was also significantly decreased (log2 ratio = −2.0, *p* = 0.0006). Collectively, these findings are consistent with coordinated suppression of stromal components associated with tumor-supportive matrix architecture, though functional assessment of matrix organization was outside the scope of this study.

Autophagy-pathway alteration and metabolic adaptation. Proteins associated with lysosomal function, autophagy, and mitochondrial metabolism were significantly altered following combination treatment. Altered abundance of Sequestosome 1 (SQSTM1/p62) (log2 ratio = 3.0, *p* < 0.00001) and Lysosomal Protein Transmembrane 4 Alpha (LAPTM4A)(log2 ratio = 2.1, *p* < 0.00001) was suggestive of dysregulated autophagic activity.

As increased p62 abundance can reflect either autophagy induction or impaired autophagic degradation (e.g., defective autophagosome–lysosome fusion), and no orthogonal flux assays (e.g., LC3-II/LC3-I turnover with and without lysosomal inhibitors or tandem mRFP-GFP-LC3 reporters) were performed, this change is reported as consistent with altered autophagic/lysosomal pathway activity rather than confirmed activation of autophagic flux. LARS2 and TMEM45A were detected exclusively in the combination-treated samples and were absent from untreated controls (*p* < 0.00001 for both); these proteins are therefore reported as presence–absence regulated features rather than quantitative fold changes. Additional increases were observed for Succinate-CoA Ligase GDP/ADP-forming subunit alpha (SUCLG1)(log2 ratio = 1.6, *p* < 0.00001), N-sulfoglucosamine Sulfohydrolase (SGSH)(log2 ratio = 1.3, *p* < 0.00001), LEPROT (log2 ratio = 1.9, *p* < 0.00001), and Ornithine Aminotransferase (OAT)(log2 ratio = 1.9, *p* < 0.00001), consistent with broad remodeling of mitochondrial metabolic pathways.

Stress response and suppression of pro-survival signaling. The combination treatment also altered proteins involved in organelle stress responses. Heat Shock Protein family E (Hsp10) member 1 (HSPE1)(log2 ratio = 2.7, *p* < 0.00001) and Mannosidase Alpha class 1B member 1 (MAN1B1)(log2 ratio = 1.0, *p* < 0.00001) were significantly increased, consistent with a signature associated with mitochondrial and endoplasmic reticulum stress pathways, though direct functional assays would be needed to confirm pathway activation. In parallel, TNIP1 was detected exclusively in the combination-treated samples and was absent from untreated controls (*p* < 0.00001), representing a presence–absence regulated feature. TNFAIP3 interacting protein 1 (TNIP1) has been reported to inhibit proliferation and promote apoptosis in other cancer contexts via C/EBPβ [53] and to regulate STimulator of Interferon Genes- STING-dependent innate immune signaling together with autophagy receptors [54], suggesting a potential role in modulating both apoptotic and innate immune signaling following combination treatment. Conversely, MAPK12 and CEP250 were undetectable in combination-treated EVs despite being consistently detected in untreated controls (MAPK12, *p* = 0.0001; CEP250, *p* = 0.0003) and are therefore reported as presence–absence regulated features rather than quantitative fold changes; this loss is consistent with a signature associated with reduced metastatic signaling and disrupted mitotic organization in the donor cells.

Summary proteomic signature. Collectively, the combined EMS2 + FTY720 treatment produced the most extensive proteomic remodeling of all treatment groups. The coordinated suppression of extracellular matrix proteins, together with alterations in autophagic, lysosomal, and mitochondrial stress-response pathway markers, and loss of metastasis- and mitosis-associated proteins, including MAPK12 and CEP250, is consistent with a greater-than-additive effect. The exclusive detection of TNIP1 following combination treatment further suggests possible concurrent modulation of apoptotic and innate immune signaling. These proteomic findings are consistent with the combined treatment engaging multiple hallmark-relevant pathways of breast cancer simultaneously; functional validation would be required to confirm these mechanistic interpretations.

## 3. Discussion

Cancer progression is increasingly recognized as a systems-level process involving metabolic dysregulation, aberrant signaling, and disruption of tissue homeostasis [55,56]. Cancer cells exhibit altered membrane potential, ion-channel activity, and calcium signaling, creating potential bioelectric vulnerabilities [27]. Low-frequency electromagnetic stimulation (EMS) may influence these processes in a frequency-dependent manner [31,57]. Both Fingolimod (FTY720), primarily known for its effects on sphingosine-1-phosphate signaling, and VER-155008, primarily characterized as an Hsc70/Hsp70 inhibitor, are pleiotropic compounds that have also been reported to affect TRPM7 activity [9,58]. Given their reported anti-cancer activity and the overexpression of TRPM7 in breast cancer, we selected these compounds to evaluate their effects in combination with electromagnetic stimulation.

While our findings are consistent with a possible contribution of TRPM7 modulation to the observed anti-proliferative and anti-metastatic protein effects, direct approaches such as TRPM7 knockdown, knockout, or neutralizing antibody-based inhibition would be needed in future studies to confirm the specific contribution of TRPM7 to these effects. In addition to target specificity, another key translational consideration for FTY720 is the divergence between its immunomodulatory plasma concentration (0.05–0.3 µM) and the higher doses (5–10 µM) required for direct in vitro anti-cancer activity [59]. To address this, we evaluated a full dose–response range (0.05, 0.5, and 5 µM) in Figure 3B. The 5 µM concentration was intentionally selected for high-throughput proteomics as a pharmacological proof-of-concept dose to ensure robust signaling network activation, permitting direct comparison against established oncology benchmarks while acknowledging the polypharmacological context of micromolar exposure.

The efficacy of our unique triple-frequency protocol, totaling just 30 min once daily, likely drives cumulative biophysical and metabolic stress while preventing the cellular adaptation common to single-frequency exposures. This mechanism differs fundamentally from traditional PEMF regimens, which rely on repeated, same-frequency exposures to disrupt stabilization between twice-daily sessions [15]. Alternating frequencies could also engage distinct cellular responses in rapid succession. These possibilities remain speculative and require direct validation through studies of membrane potential, mitochondrial function, and cellular stress pathways.

Although an initial exposure experiment showed no significant difference from standard medium and sham exposure controls, contemporaneous sham-exposed controls were not included in all principal experiments, including proteomic studies. Thus, subtle effects associated with device placement cannot be completely excluded, and future studies should incorporate matched sham controls throughout.

Bliss independence analysis revealed greater-than-additive suppression of invasion-associated protein levels in EVs (MMP-2, MMP-19, VEGF-C, and MAPK12) that did not correlate with cell proliferation assays. This divergence occurs because MTS assays measure general cell viability, whereas our proteomic data captured early, compartment-specific signaling alterations in secreted extracellular vesicles. Furthermore, our single 30 min EMS2 exposure session may be insufficient to manifest a macroscopic proliferative phenotype, unlike the repeated or fractionated exposure regimens utilized in other studies to achieve cumulative anti-proliferative effects [31,33]. These results demonstrate that combined treatment preferentially reshapes invasive signaling ahead of cytotoxicity, highlighting the sensitivity of proteomic profiling. Functional validation via invasion and migration assays remains necessary to confirm these anti-metastatic effects.

Our EVs characterization was limited to detection of the tetraspanin markers CD9, CD63, and CD81 by LC-MS/MS following exoEasy-based isolation. Consistent with MISEV2023 guidelines [60], comprehensive EV characterization would additionally include particle concentration and size distribution analysis (e.g., nanoparticle tracking analysis), morphological assessment (e.g., TEM or cryo-EM), a broader panel of EV-associated and non-EV/contaminating markers, and EV yield normalized to cell number or conditioned-medium volume. These additional analyses were not performed in the current study and represent an important direction for future work to more rigorously define the isolated vesicle population and assess potential co-isolation of non-vesicular proteins.

Paclitaxel produced an expected drug-associated EV proteomic signature, supporting the ability of the approach to detect treatment-associated molecular remodeling [61].

EMS2 exposure was associated with changes in metabolic, redox, vesicular-trafficking, and centrosomal proteins, consistent with cellular adaptation and reduced proliferative capacity. The enrichment of metabolic and redox-associated proteins (MPST, AUH, and IMPA2) alongside strong induction of vesicle trafficking components (EHD3, LGALS7, and STC1) suggests activation of vesicle-driven adaptation program. Simultaneous downregulation of key structural and regulatory proteins, including CEP250, CHAMP1, and RB1CC1, indicates disruption of centrosomal integrity, chromosome organization, and autophagy-associated regulatory networks.

It is important to emphasize that the proteomic profiles associated with altered cell-cycle progression, mitochondrial stress, ER stress, and immune modulation observed in this study represent molecular signatures rather than verified phenotypic alterations. While these alterations provide a valuable hypothesis-generating framework for the downstream cellular response to EMS2, they remain tentative until corroborated by targeted functional assays. Although these changes are compatible with stress-associated altered cell-cycle progression and senescence-like state, functional assays such as SA-β-gal staining, extended cell-cycle analysis, and assessment of p53/p21 or p16/Rb signaling are required to establish cellular senescence. In addition, live-cell imaging or biochemical cell-cycle markers are required in future studies to definitively confirm whether these structural anomalies stem from a true arrest at a specific mitotic stage.

While EMS2 treatment led to prominent cell enlargement, morphology alone is insufficient to establish a true mitotic block. Future work utilizing time-lapse microscopy, flow cytometric cell-cycle analysis, or phospho-histone H3 tracking will be essential to precisely define how EMS2 impacts the cell division machinery.

Consistent with previous reports showing FTY720 suppresses breast cancer stem cell expansion via PP2A activation [43] and disrupts phosphoinositide-dependent trafficking and nutrient metabolism in other cancer models [62], our proteomic data extend these findings by showing upregulation of ribosomal proteins (RPS29, RPS25, and RPS15) together with oxidative stress-associated factors, suggesting disruption of translational homeostasis and activation of compensatory stress-response pathways specific to the extracellular vesicle compartment. Concurrent downregulation of ECM-associated proteins, including Tenascin-C and SNED1, indicates attenuation of structural support systems associated with invasion and metastatic niche maintenance proteomic levels in EVs. However, the persistence of antioxidant responses, including SRXN1 induction, suggests that FTY720 alone does not fully overcome adaptive stress resistance mechanisms.

Our proteomic analysis revealed a significant accumulation of SQSTM1/p62 following treatment. While p62 is a classic marker utilized in autophagy monitoring, changes in its steady-state protein levels alone cannot conclusively demonstrate autophagic flux. Elevated p62 can indicate either an upregulation of autophagosome synthesis or a disruption in downstream lysosomal degradation pathways. Distinguishing between these mechanisms would require formal flux validations utilizing lysosomal inhibitors such as bafilomycin A1 or tandem fluorescent LC3 reporter assays. Therefore, our current observations should be interpreted as a generalized dysregulation of autophagic activity rather than a confirmed activation of flux.

Dual treatment EMS2 + FTY720 produced a more coordinated proteomic response than either treatment alone. It markedly suppressed extracellular matrix (ECM) and stromal protein levels, consistent with disruption of proteins in EVs associated with invasion and metastasis. Concurrently, undetectable levels of MAPK12 and CEP250 in dual treatment and only partially reduced by either single agent suggests a more pronounced disruption of signaling and mitotic stability pathways under combination treatment. Notably, the exclusive detection of TNIP1 under combination therapy highlights a potentially important regulatory node. TNIP1 has been reported to restrict STING-mediated innate immune signaling in cooperation with autophagy receptors [63]. Because TNIP1 is normally targeted for autophagic degradation following TBK1-mediated phosphorylation of its LC3-interacting region (LIR) [64], its selective retention in EVs proteomics under combination treatment may reflect altered proteostatic regulation. This molecular signature is consistent with a coordinated stress response involving innate immune and autophagic pathways, which may increase susceptibility to cell death; functional validation would be needed to confirm this outcome.

## 4. Materials and Methods

### 4.1. Reagents and Cell Culture for In Vitro Assays

The cell culture procedure was performed as described previously [24]. Human mammary epithelial cells were purchased from Gibco (Thermo Fisher Scientific, Waltham, MA, USA) and cultured according to the manufacturer’s recommendations. The MDA-MB-231 breast cancer cell line was obtained from ATCC and cultured in DMEM 10% FBS. ARM-G and ARM-X was originally derived from patient breast cancer tissue, never passaged in mice, characterized, and subsequently adapted to serum-free medium with supplements, as previously described [24]. The cell line has not been deposited in a public repository to date. Prior to use, all cell lines were STR authenticated by the provider. For EVs isolation experiments, ARM-G cells were cultured in phenol red-free Ham’s F12 medium (Caisson Labs/Plant Cell Technology, Smithfield, UT, USA) without FBS. All procedures were carried out under aseptic conditions as previously described. Cells were incubated at 37 °C with 5% CO_2_. After thawing, all cell lines were used for no more than five passages from the original frozen stock. The absence of mycoplasma contamination in all cell lines was confirmed using the MycoAlert^®^ Mycoplasma Detection Kit (reference LT07-318, Lonza™, Basel, Switzerland). Cells were grown to a confluence of 70–90% before being split and prepared at the required concentrations for in vitro assays.

For the MTS assay, 10–15 × 10^3^ cells per well were seeded in a 96-well plate, and the assay was performed on day 2 post-treatment. Cell numbers were determined using an EVE™ automated cell counter (NanoEnTek, Seoul, South Korea). The TRPM7 inhibitors Ver-155008 [58,65] and FTY720 [23,40] were purchased from Sigma-Aldrich (St. Louis, MO, USA).

### 4.2. Electromagnetic Stimulation Device and Exposure Conditions

#### 4.2.1. Rationale for Device Design and Description

The IteraCoil device used for electromagnetic stimulation (EMS) in the present study was the same device previously described and characterized in detail [17]. Electromagnetic stimulation (EMS) was delivered using the IteraCoil device, a custom-manufactured dual-solenoid apparatus (A. Tsaghikian, PhD). The device consists of two independently operable coils mounted on a shared platform with an integrated ventilation system to maintain coil temperature below 37 °C during operation.

This coil-based, non-contact approach was selected specifically to avoid electrochemical reactions at the electrode–tissue interface and the need for molecular scaffolds, conductive networks, or salt bridges required by direct-electrode stimulation methods [66,67,68,69,70]. Copper was selected for high electrical conductivity in the driving coil and iron for high magnetic permeability in the field-generating coil.

Each IteraCoil comprises two nested solenoids. The primary solenoid was formed by winding annealed iron wire (mild steel, 1.0 mm diameter, 1600 mm length; GoodFellow USA) around a 3D-printed acrylonitrile butadiene styrene (ABS) spool. The spool was cylindrical, 80 mm in length, with a 12 mm inner diameter and 14 mm outer diameter and was sized to closely accommodate one standard 10 × 80 mm laboratory test tube positioned coaxially within the coil for the full length of the winding. The secondary solenoid was formed by tightly winding enameled copper wire (0.4 mm diameter, 15.6 m length, total resistivity 2.12 Ω; Amazon.com) without gaps around the iron (primary) solenoid.

To prevent electromagnetic crosstalk between the two coils and permit simultaneous, independent testing of two conditions, each coil was individually wrapped in mu-metal magnetic shielding foil (0.012” × 8” × 12”; Magnetic Shield, Bensenville, IL, USA). All electronic control components were housed within the platform [17].

#### 4.2.2. Signal Generation and Waveform

The device delivered a quasi-rectangular alternating electric current to the copper (secondary) solenoid of each coil independently. Frequency (range: 1–10,000 Hz) and duty cycle (range: 1–100%) were user-configurable via the device dashboard for each coil separately, enabling two different stimulation protocols to be run concurrently.

When alternating current was applied to the copper wire, it generated an alternating magnetic field within the iron (primary) solenoid, which, in turn, induced an alternating electric field within the cylindrical space of the iron coil where the culture vessel was positioned. Because the lines of magnetic force are predominantly confined within the iron wire itself, field characterization was performed by measuring the induced electric field within the sample space, as this represents the field to which cells were directly exposed.

Copper was selected for the driving (secondary) solenoid on the basis of its high electrical conductivity, and iron was selected for the field-generating (primary) solenoid on the basis of its high magnetic permeability. This inductive, non-contact design was adopted to avoid electrochemical reactions at the point of contact between electrodes and cells/tissue and to eliminate the need for conductive molecular scaffolds, networks, or salt bridges required by direct-electrode stimulation methods [66,67,68,69,70].

#### 4.2.3. Field Characterization

Prior to the start of experiments, the electric field output of the IteraCoil device was characterized empirically across the four-culture media and buffers used in this study: Dulbecco’s Modified Eagle Medium (DMEM), phosphate-buffered saline (PBS), Roswell Park Memorial Institute medium (RPMI), and normal saline.

A glass tube matching the material and dimensions of the test tubes used in the experiments was fitted with electrodes inserted at both ends and placed within the coil, filled individually with each medium/buffer. An empty tube served as a control. The electrodes were connected to a digital oscilloscope (Hantek DSO5072P, 70 MHz bandwidth; Hantek Electronic Co., Ltd., Qingdao, China) to record the electric potential generated within each medium [17].

Each medium was tested at three input frequencies (50, 500, and 1000 Hz) and three duty cycle settings (20%, 50%, and 80%), yielding a total of 36 (frequency × duty cycle × medium) combinations. For each combination, a quasi-rectangular input signal was applied to the coil and the resulting electric potential in the medium was recorded.

Across all conditions tested, the induced electric field within each medium consistently matched the frequency, duty cycle, and quasi-rectangular waveform shape of the input signal applied to the copper coil. Input peak-to-peak voltage ranged from 13.2 to 16.8 V. Output peak-to-peak voltage within the media ranged from 92 to 116 mV (1.15–1.45 mV/mm), and output RMS voltage ranged from 8 to 20 mV (0.10–0.25 mV/mm).

Magnetic flux density at the sample position and current draw through the coil were not directly measured in this study; characterization was instead based on the induced electric field within the sample space, which is the parameter most directly relevant to the cells given the confinement of magnetic flux lines within the iron core. Direct magnetic field measurement (e.g., via Hall-probe or gaussmeter) at the sample position was not performed and is noted as a limitation.

The electric field values reported above represent an axial average across the length of the tube, obtained from electrodes positioned at each end, rather than a spatial map of field strength within the vessel. Within-tube spatial uniformity was not separately characterized, and this is noted as a limitation of the present field characterization.

#### 4.2.4. Temperature Monitoring

Coil temperature was characterized prior to experimental use to confirm that stimulation did not raise culture temperature above physiological range. The temperature of the inner wall of each coil’s sample space was continuously monitored for 2 h using a 1-Wire programable digital thermometer (DS18B20) (Maxim Integrated, San Jose, CA, USA) with the device powered on and the ventilation system active. Representative operating settings were used for this characterization: the left coil was set to 50 Hz with a 50% duty cycle and the right coil was set to 1000 Hz with a 50% duty cycle. Temperature fluctuated between 30.2 °C and 36.8 °C over the 2 h monitoring period, remaining below 37 °C throughout [17].

Continuous temperature monitoring during the EMS1, EMS2, and EMS3 exposure protocols themselves was not performed, as the presence of the thermometer probe risked interference with the applied electromagnetic field. The pre-experiment characterization above was therefore used as a proxy for in-experiment thermal conditions at matched device settings.

#### 4.2.5. Vessel Positioning

Standard 10 × 80 mm laboratory test tubes containing cell cultures were placed within the inner space of each IteraCoil, which was dimensioned (12 mm inner diameter; 80 mm length) to position the vessel coaxially with the coil axis and centered along its full winding length, with no intentional air gap or lateral offset.

#### 4.2.6. Protocols of EMS and FTY720 Treatment of Breast Cancer Cell Lines

Rationale for Candidate Frequency Selection

The specific candidate frequencies evaluated in this study—396 Hz, 417 Hz, 528 Hz, and 285 Hz for EMS1/EMS2 and 21 Hz for EMS3—were selected based on prior reports demonstrating bioelectric or antiproliferative effects of low-frequency electromagnetic stimulation at or near these values when applied as single, continuous frequencies [57,71,72]. To further validate this selection, preliminary testing was conducted using standard baseline frequencies (100 Hz, 200 Hz, 500 Hz, and 1000 Hz) for 30 min; however, no anti-cancer activity was detected at these intervals.

While these prior studies characterized the biological effects of individual frequencies strictly in isolation, the present study extended this paradigm. Using the previously reported single-frequency values as an empirical starting point rather than as pre-validated combinatorial formulas, we evaluated whether sequential, combinatorial exposure to multiple frequencies in a defined order (EMS1 and EMS2) or a single extended exposure (EMS3) could produce enhanced antiproliferative effects.

EMS Protocol Definitions:

EMS protocols used in the present study consisted of the defined frequency sequences designated EMS1, EMS2, and EMS3. The individual frequencies, order of frequency application, duration of each frequency exposure, total exposure duration, and duty cycle of 50% are provided in the corresponding experimental descriptions below.

EMS1: 396 Hz for 10 min → 417 Hz for 10 min → 528 Hz for 10 min;

EMS2: 396 Hz for 10 min → 285 Hz for 10 min → 528 Hz for 10 min;

EMS3: 21 Hz for 30 min (single exposure).

Two-Stage Screening and Confirmation Design

EMS1, EMS2, and EMS3 were evaluated in two sequential stages using distinct cell lines and culture conditions, in order to distinguish exploratory screening from independent confirmatory testing.

Stage 1: Exploratory screening (ARM-G cells). EMS1, EMS2, and EMS3 were first evaluated in ARM-G cells cultured in serum-free medium. All three protocols demonstrated antiproliferative activity in this exploratory screening stage.

Stage 2: Independent confirmation (MDA-MB-231 cells). EMS1, EMS2, and EMS3 were subsequently tested independently in a second cell line, MDA-MB-231, cultured under different conditions (10% serum). In this independent test, only EMS2 retained suppressive activity; EMS1 and EMS3 did not. Because this result was obtained in a distinct cell line and culture condition from the initial ARM-G screening, the antiproliferative effect of EMS2 reported in this manuscript reflects independent confirmation rather than a result derived from the same dataset used for its selection.

Outcome Assessment

Cell viability was assessed on day 2 by counting the cell number or MTS assay using the CellTiter 96^®^ AQueous One Solution Cell Proliferation Assay (Cat. No. G3580; Promega, Madison, WI, USA) according to the manufacturer’s instructions.

For combination treatment, FTY720 was prepared from a 10 mM stock solution in DMSO and diluted 1:2000 in culture medium to a final concentration of 5 μM. FTY720 was added to the culture medium at the onset of EMS2 exposure (396 Hz–10 min; 285 Hz–10 min; 528 Hz–10 min; 30 min total) and remained in the medium for the full 48 h incubation period before subsequent analyses were performed. As a negative control, cells were treated with vehicle (DMSO diluted 1:2000 in culture medium) alone, matching the DMSO concentration used in the FTY720 treatment condition. Cell morphology was assessed by phase-contrast microscopy using a Nikon microscope (Nikon, Tokyo, Japan) at 200× magnification. Images were captured using Swift Imaging 3.0 software (Swift Optical Instruments/Motic Swift Line, Schertz, TX, USA).

### 4.3. Bliss Independence Model

Bliss independence was assessed using the percentage of control signal remaining for each protein following treatment with EMS2 alone, FTY720 alone, or the combination, where lower values indicate greater inhibition relative to untreated control. The expected combined signal remaining (E_Bliss) was calculated as the product of the individual remaining fractions: E_Bliss = A × B, where A and B represent the percentage of control signal remaining after EMS2 and FTY720 treatment, respectively. Greater inhibition than predicted by E_Bliss was defined as an observed combination value lower than E_Bliss (i.e., greater inhibition than predicted by independent action); antagonism was defined as an observed value higher than E_Bliss.

### 4.4. Extracellular Vesicle (EVs) Isolation and Purification

For extracellular vesicle (EVs) extraction, ARM-G cells (seeded at 1–1.5 × 10^6^ cells) subjected to the treatments described above were cultured in triplicate in T-75 cm^2^ flasks containing 15 mL of serum-free medium with supplements. After 48 h, conditioned medium from each flask was collected separately and subjected to initial centrifugation at 300 *g* for 10 min at 4 °C to pellet residual cells. The resulting supernatants were carefully transferred to fresh centrifuge tubes, ensuring the pellet remained undisturbed, and subsequently passed through a 0.45 µm filter to remove remaining large particulate debris. EVs were isolated from the clarified supernatants using the exoEasy Maxi Kit (Qiagen, Hilden, Germany) in strict accordance with the manufacturer’s instructions. EVs were eluted in 350 μL of elution buffer. Purified EVs preparations were either utilized immediately for downstream analyses or stored at −80 °C and submitted to the Rutgers University Proteomics Core Facility for LC-MS/MS analysis.

Extracellular vesicle identity was confirmed by LC-MS/MS detection of the canonical exosomal markers CD81, CD63, and CD9 across all treatment conditions, with spectral counts reported in Supplementary Table S3 EVs were isolated from *n* = 3 independent biological preparations (independent cultures) per condition. Each of the 3 independent preparations was subjected to a single LC-MS/MS run (i.e., 3 independent biological replicates per condition, without technical injection replicates).

### 4.5. Sample Preparation for Proteomic Analysis: SP3 (Single-Pot Solid-Phase-Enhanced Sample Preparation) Digestion Protocol for Efficient Protein Extraction and Digestion

Ten micrograms of sample were diluted in 50 µL of 2X lysis buffer (2% SDS, 50 mM HEPES, pH 8.0, 50 mM EDTA). Disulfide bonds were reduced by the addition of 5 mM DTT for 30 min at 60 °C. Free cysteines were subsequently alkylated with 20 mM iodoacetamide for 1 h at room temperature in the dark. Samples were then processed using the SP3 bead-based digestion method as described by Hughes et al. [73]. Proteolytic digestion was carried out with sequencing-grade trypsin (Cat. #90058,Thermo Scientific, Waltham, MA, USA) in 100 mM ammonium bicarbonate containing 2 mM CaCl_2_ and incubated overnight at 37 °C.

Following digestion, peptides were acidified with formic acid and concentrated to approximately 20 µL using a vacuum concentrator. Subsequently, 100 µL of 50% acetonitrile containing 0.1% TFA was added, and the samples were incubated at room temperature for 30 min. Samples were centrifuged at 25,000× *g* for 10 min, and the supernatant was collected and further dried in a vacuum concentrator to a volume of less than 10 µL. The final volume was adjusted to 20 µL with 0.1% TFA.

### 4.6. Liquid Chromatography–Tandem Mass Spectrometry (LC-MS/MS)

Peptides were analyzed by nano-LC-MS/MS using a Vanquish Neo nanoLC system (Thermo Fisher Scientific, Waltham, MA, USA) coupled to a timsTOF HT mass spectrometer (Bruker Daltonics, Bremen, Germany). Samples were directly loaded onto a PepSep Ultra C18 column (75 µm × 25 cm, Bruker, Bremen, Germany) and separated at a flow rate of 300 nL/min using a segmented linear gradient: 2–12% solvent B over 16 min, 12–20% B over 16 min, and 20–32% B over 12 min (solvent A: 0.2% formic acid in water; solvent B: 0.2% formic acid in acetonitrile).

For DIA-PASEF acquisition, precursor ions with m/z values between 300 and 1200 were analyzed using eight DIA-PASEF scans per cycle, each consisting of three quadrupole isolation windows and 24 ion mobility steps spanning a range of 0.7–1.3 (1/K_0_). Variable isolation window widths of 36–41 Th were applied for each ion mobility step. The ramp time and accumulation time were both set at 85 ms to make the ramp rate of 10.97 HZ.

### 4.7. Proteomic Data Analysis

Raw data were searched using a predicted spectral library generated from a UniProt human-reviewed FASTA database for direct DIA analysis in Spectronaut version 20 (Biognosys AG, Schlieren, Switzerland) with recommended default settings. Only protein groups with a posterior error probability (PEP) score < 0.05 in each run and a protein-group identification q-value < 0.01 were retained. Protein-group quantification was normalized across the experiment using the MaxLFQ method, and protein groups identified by a single precursor were excluded from analysis.

Proteomic analyses were performed using three independent biological replicates per condition. For pairwise comparisons, a protein group was required to have quantified MaxLFQ values in at least 2 of the 3 biological replicates in at least one of the two compared treatment groups; remaining missing values for protein groups meeting this criterion were imputed as zero.

No additional normalization was applied beyond MaxLFQ. Pairwise statistical comparisons were performed using a modified QuasiSeq approach based on a quasi-Poisson generalized linear model implemented in edgeR (version X.X.X), using a previously published R script, using a previously published R script [74]. Resulting *p*-values were adjusted for multiple comparisons using the Benjamini–Hochberg procedure, and proteins were considered differentially abundant at a false-discovery-rate (adjusted *p*-value/*q*-value) threshold of <0.01. This differential-abundance threshold is distinct from the protein-group identification *q*-value (<0.01) described above, which reflects identification-level confidence rather than differential-expression statistical significance.

### 4.8. Statistical Analyses

For pairwise comparisons of data (e.g., MTS and cell count proliferation assays), two-tailed unpaired Student’s *t*-tests were used to compare means between independent groups. *p* values are presented in the corresponding figures, with significance denoted as * *p* < 0.05, ** *p* < 0.01, and *** *p* < 0.001 relative to untreated controls unless otherwise indicated.

## 5. Conclusions

This study demonstrates that combined FTY720 and EMS2 treatment induces coordinated proteomic remodeling in breast-cancer-derived extracellular vesicles (EVs), reflecting complementary mechanisms of action. FTY720 triggers ribosomal stress, activates oxidative responses, and reduces levels of extracellular matrix (ECM)-associated proteins, while EMS2 drives metabolic reprograming, alters vesicular trafficking, and disrupts centrosomal and structural regulatory networks, producing a proteomic signature consistent with a senescence-like state.

The combination produced a more extensive proteomic shift in EVs than either treatment alone. This shift is characterized by the pronounced downregulation of stromal and ECM-associated proteins (including collagen components and MATN3) and undetectable levels of MAPK12 and CEP250 proteins in EVs—both of which were only partially reduced by either single agent. These alterations indicate a marked escalation of proteomic disruption under combination treatment, alongside concurrent activation of autophagy- and stress-associated pathways.

Bliss independence analysis further identified a greater-than-additive suppression of the invasion- and metastasis-associated proteins MMP-2, MMP-19, VEGF-C, and MAPK12 in EVs. This exploratory finding is consistent with a coordinated molecular interaction between the two treatments. Notably, this greater-than-additive suppression at the EV proteomic level was not accompanied by a corresponding pattern in cell proliferation, suggesting that the combination may preferentially alter levels of proteins associated with invasive and metastatic signaling ahead of measurable changes in proliferative capacity. Given that EMS2 was administered as a single triple-frequency exposure session (10 min per frequency, 30 min total), repeated or fractionated dosing regimens may be required to translate these molecular proteomic changes into measurable proliferative effects.

The selective enrichment of TNIP1 identified via EV proteomic sequencing reveals a coordinated innate immune and autophagic stress response, positioning this molecular signature as a potential biomarker of treatment-induced tumor cell susceptibility to death. While these findings are derived from in vitro and EVs proteomic analyses, they provide a strong rationale for further mechanistic and in vivo studies—including repeated EMS2 dosing regimens and functional invasion/migration assays—to determine whether this combinatorial molecular remodeling translates into a measurable functional and therapeutic benefit.

## Figures and Tables

**Table 1:** Bliss independence analysis of EMS2 and FTY720 combined effects on MMP-2, MMP-19, VEGF-C, and MAPK12 levels in ARM-G-derived EVs. Values represent the percentage of control signal remaining following each treatment; lower values indicate greater suppression. Interaction classifications reflect deviation from the Bliss-predicted additive expectation (see Section 4).

Protein	A (EMS2)	B (FTY)	E_Bliss	Observed Combination	Interaction
MMP-2	69%	100%	69.00%	34%	Greater-than-additive suppression
MMP-19	70%	94%	65.80%	39%	Greater-than-additive suppression
VEGF-C	81%	99%	80.20%	52%	Greater-than-additive suppression
MAPK12	30%	43%	12.90%	0.50%	Greater-than-additive suppression

## Data Availability

The data presented in this study are available on request from the corresponding author due to intellectual property protection and a pending patent application.

## Supplementary Materials

The following supporting information can be downloaded at https://www.mdpi.com/article/10.3390/ph19091399/s1, Figure S1: Validation of the sham control for electromagnetic stimulation experiments; Table S1: Inhibition of breast cancer cell proliferation following treatment with paclitaxel or electromagnetic stimulation (EMS) (corresponding to Figure 1); Table S2: Effect of combined treatment with FTY720 (at different concentrations) and EMS2 on ARM-G breast cancer cell proliferation (corresponding to Figure 3); Table S3: MaxLFQ intensities (mean ± SD, *n* = 3) determined by LC-MS/MS for canonical exosomal markers CD81, CD63, and CD9 across treatment conditions, confirming exosome identity in ARM-G-derived extracellular vesicles following each treatment.

## Abbreviations

The following abbreviations are used in this manuscript:

TNBC	Triple-Negative Breast Cancer
EMS	Electromagnetic Stimulation
EVs	Extracellular Vesicles
TRPM7	Transient Receptor Potential Melastatin 7
TME	Tumor Microenvironment
TTFields	Tumor Treating Fields
PEMF	Pulsed Electromagnetic Field
HMEC	Human Mammary Epithelial Cells
