# Supplementary material for: Triple-Frequency Electromagnetic Stimulation Combined with Fingolimod Reduces Breast Cancer Cell Proliferation and Metastasis-Associated Extracellular Vesicle Protein Levels
Source: Pharmaceuticals (Basel). 2026 Sep 4;19(9):1399. doi: 10.3390/ph19091399 (PMC13610236; doi:10.3390/ph19091399)
Supplement: Supplementary file 1 [file pharmaceuticals-19-01399-s001.zip › Table S1-N.pdf]

Table S1. Inhibition of breast cancer cell proliferation following treatment with paclitaxel or electromagnetic stimulation (EMS) (corresponding to Figure 1).

ARM-G, ARM-X, and MDA-MB-231 cells were treated with paclitaxel as a positive control (PC) or different EMS frequencies (EMS1, EMS2, EMS3), corresponding to Figure 1. Values represent the mean percentage of cell count relative to untreated control (mean ± SD, n = 3); lower values indicate greater growth inhibition.

Cell line	Paclitaxel (PC)	EMS1	EMS2	EMS3
ARM-G	28.2 ± 4.9%	47.8 ± 9.8%	22.5 ± 9.7%	56.3 ± 10.4%
ARM-X	55.9 ± 5.8%	43.7 ± 3.2%	57.5 ± 1.7%	64.3 ± 2.3%
MDA-MB-231	69.8 ± 11.9%	88.0 ± 7.3%	58.7 ± 2.8%	92.1 ± 13.7%
