# Supplementary material for: Triple-Frequency Electromagnetic Stimulation Combined with Fingolimod Reduces Breast Cancer Cell Proliferation and Metastasis-Associated Extracellular Vesicle Protein Levels
Source: Pharmaceuticals (Basel). 2026 Sep 4;19(9):1399. doi: 10.3390/ph19091399 (PMC13610236; doi:10.3390/ph19091399)
Supplement: Supplementary file 1 [file pharmaceuticals-19-01399-s001.zip › Table S2-N.pdf]

Table S2. Effect of combined treatment with FTY720 (at different concentrations) and EMS2 on ARM-G breast cancer cell proliferation (corresponding to Figure 3). Cells were treated with EMS2 alone, FTY720 alone (at varying concentrations), or the combination. Values represent the mean percentage of untreated control cell count (mean ± SD, n = 3); lower values indicate greater growth inhibition.

Treatment Condition	NC	PC	FTY-5μM	FTY-0.5μM	FTY-0.05μM	EMS2	EMS2+FTY 5μM	EMS2+FTY 0.5μM	EMS2+FTY 0.05μM
% of Control	100±5%	82±6%	74±3%	84±9%	85±10%	82±10%	72±8%	80±12%	89±13%
