# Supplementary material for: Triple-Frequency Electromagnetic Stimulation Combined with Fingolimod Reduces Breast Cancer Cell Proliferation and Metastasis-Associated Extracellular Vesicle Protein Levels
Source: Pharmaceuticals (Basel). 2026 Sep 4;19(9):1399. doi: 10.3390/ph19091399 (PMC13610236; doi:10.3390/ph19091399)
Supplement: Supplementary file 1 [file pharmaceuticals-19-01399-s001.zip › Table S3--N.pdf]

Table S3. MaxLFQ intensities (mean ± SD, n = 3) determined by LC-MS/MS for canonical exosomal markers CD81, CD63, and CD9 across treatment conditions, confirming exosome identity in ARM-G-derived extracellular vesicles following each treatment.

Protein	NC	PC	FTY	EMS2	EMS2+FTY
CD81	$2.5{\times}10^4 \pm 0.57{\times}10^4$	$1.9{\times}10^4 \pm 0.19{\times}10^4$	$1.6{\times}10^4 \pm 0.19{\times}10^4$	$2.2{\times}10^4 \pm 0.27{\times}10^4$	$2.2{\times}10^4 \pm 0.56{\times}10^4$
CD63	$2.0{\times}10^4 \pm 0.72{\times}10^4$	$1.8{\times}10^4 \pm 0.18{\times}10^4$	$1.9{\times}10^4 \pm 0.18{\times}10^4$	$2.5{\times}10^4 \pm 0.48{\times}10^4$	$4.1{\times}10^4 \pm 0.60{\times}10^4$
CD9	$2.0{\times}10^4 \pm 0.43{\times}10^4$	$2.3{\times}10^4 \pm 0.23{\times}10^4$	$2.4{\times}10^4 \pm 0.23{\times}10^4$	$2.9{\times}10^4 \pm 0.24{\times}10^4$	$2.9{\times}10^4 \pm 0.91{\times}10^4$

Note: *Values are expressed as* MaxLFQ. *NC = negative control; PC = positive control; FTY = FTY720 treatment; EMS2 = EMS2 treatment; EMS2+FTY = combined treatment.*
